# Reduced task-related functional connectivity during a set-shifting task in unmedicated early-stage Parkinson’s disease patients

**DOI:** 10.1186/s12868-016-0254-y

**Published:** 2016-05-18

**Authors:** Corine C. de Bondt, Niels J. H. M. Gerrits, Dick J. Veltman, Henk W. Berendse, Odile A. van den Heuvel, Ysbrand D. van der Werf

**Affiliations:** Department of Anatomy and Neurosciences, VU University Medical Center (VUmc), Van der Boechorststraat 7, 1081 BT Amsterdam, The Netherlands; Netherlands Institute for Neuroscience, An Institute of the Royal Netherlands Academy of Arts and Sciences, Amsterdam, The Netherlands; Department of Neurology, VU University Medical Center (VUmc), Amsterdam, The Netherlands; Department of Psychiatry, VU University Medical Center (VUmc), Amsterdam, The Netherlands; Neuroscience Campus Amsterdam (NCA), Amsterdam, The Netherlands

**Keywords:** Parkinson’s disease, Task-related functional connectivity, Set-shifting, Functional magnetic resonance imaging, Compensation

## Abstract

**Background:**

Patients with Parkinson’s disease (PD) often suffer from cognitive impairments, including set-shifting deficits, in addition to the characteristic motor symptoms. It is hypothesized that the striatal dopamine depletion leads to a sub-optimal functional connectivity between task-related brain areas and consequently results in impaired task-performance. In this study, we aimed to examine this hypothesis by investigating the task-related functional connectivity of brain areas that are believed to be involved in set-shifting, such as the dorsolateral prefrontal cortex (DLPFC), posterior parietal cortex (PPC) and the superior frontal gyrus (SFG), during a set-shifting task. We obtained functional imaging data from 18 early-stage PD patients and 35 healthy controls, matched at the group level, using a newly developed rule-based set-shifting task that required participants to manually respond to arrow stimuli based on their location on the screen of their direction.

**Results:**

We found that early stage PD patients, compared with controls, showed (1) a decrease in positive coupling between the left DLPFC and the right insular cortex, and the right SFG and anterior cingulate cortex, (2) an increase in negative coupling between the right SFG and the anterior cingulate cortex, primary motor cortex, precuneus, and PPC, and (3) an increase in negative coupling between the left DLPFC and the left and right SFG. These results indicate that important task-related areas of PD patients have decreased functional connectivity with task-related regions and increased connectivity with task-unrelated areas.

**Conclusions:**

The disruption of functional connectivity in early stage PD patients during set-shifting reported here is likely compensated for by the local hyperactivation we reported earlier, thereby forestalling behavioural deficits.

## Background

Parkinson’s disease (PD) is a neurodegenerative disorder characterized by, among others, loss of dopamine neurons of the substantia nigra *pars compacta* [1]. This degeneration results in a dopamine depletion within the frontal-striatal circuits, leading to hypo-excitation of cortical areas, including the frontal lobes [2, 3]. As a consequence, characteristic clinical motor symptoms arise, such as bradykinesia, resting tremor, and rigidity. Besides these motor symptoms, patients with PD often suffer from non-motor symptoms, such as sleep disturbances, autonomic problems, neuropsychiatric symptoms (e.g., depression, hallucinations, impulse control disorders, and anxiety), and cognitive dysfunction [4, 5]. The latter includes attention problems, visuospatial deficits, and executive dysfunctions, such as set-shifting difficulties [6–8] resulting in cognitive rigidity.

Set-shifting is defined as a mental process that is necessary to switch attention from one action or rule to another action or rule [9]. Although numerous studies employed the Wisconsin Card Sorting Task (WCST) [10] to investigate set-shifting, more recent investigations have shown that task performance on the WCST not only depends on set-shifting capacities, but also on other cognitive constructs, such as working memory [11], concept formation, and rule learning [12]. Also, the use of dopaminergic medication influences task-performance on set-shifting tasks in patients with PD [13]. These potential confounding factors might have resulted in spurious findings in behavioural performance and neuronal activation, thereby providing an inaccurate view on set-shifting in PD: various authors have noted set-shifting difficulties in PD patients in association with cortical and subcortical activation differences as compared to controls (e.g. [14]), but it has been noted that these are hard to disentangle from the effects of other cognitive deficits, motor deficits, effects of dopaminergic medication and/or withdrawal, and mood [13]. We recently developed a new set-shifting task with a higher construct validity and used this task to study set-shifting in early stage PD patients, who were not using dopaminergic medication [15]. We showed equal behavioural performance across groups, but during task performance PD patients, compared with controls, showed hyper-activation of the bilateral PPC and right SFG and hypo-activation of the right ventrolateral prefrontal cortex (VLPFC). We concluded that the hypo-activation of the VLPFC was compensated for by the hyper-activation of the PPC and other task-related brain areas, thereby forestalling behavioural deficits.

Neuro-imaging studies have suggested that striatal dopamine depletion results in a decreased synchronization (i.e. functional connectivity) between brain areas [16, 17], both during rest [18–21] and task performance [22, 23]. We recently found supporting evidence for this hypothesis, based on fMRI data from the same patients and controls as the present study during a working memory paradigm [24]. We found that early stage PD patients hyper-activated task-related areas during working memory processing, but showed a reduced inter-regional connectivity. We interpreted the hyper-activation as compensation for the reduction in task-related network connectivity.

In order to gain more insight into the changes in task-related functional connectivity in early stage PD in relation to set-shifting, and relate them to our preceding findings concerning changes in task-related activity, we investigated the task-related functional connectivity of the bilateral DLPFC, bilateral SFG, and bilateral PPC, using psycho-physiological interaction analysis (PPI) [25]. We hypothesized that the functional connectivity between task-related brain areas would be decreased in early stage PD patients compared with matched healthy controls.

## Results

### Functional connectivity DLPFC

**Fig. 1:**
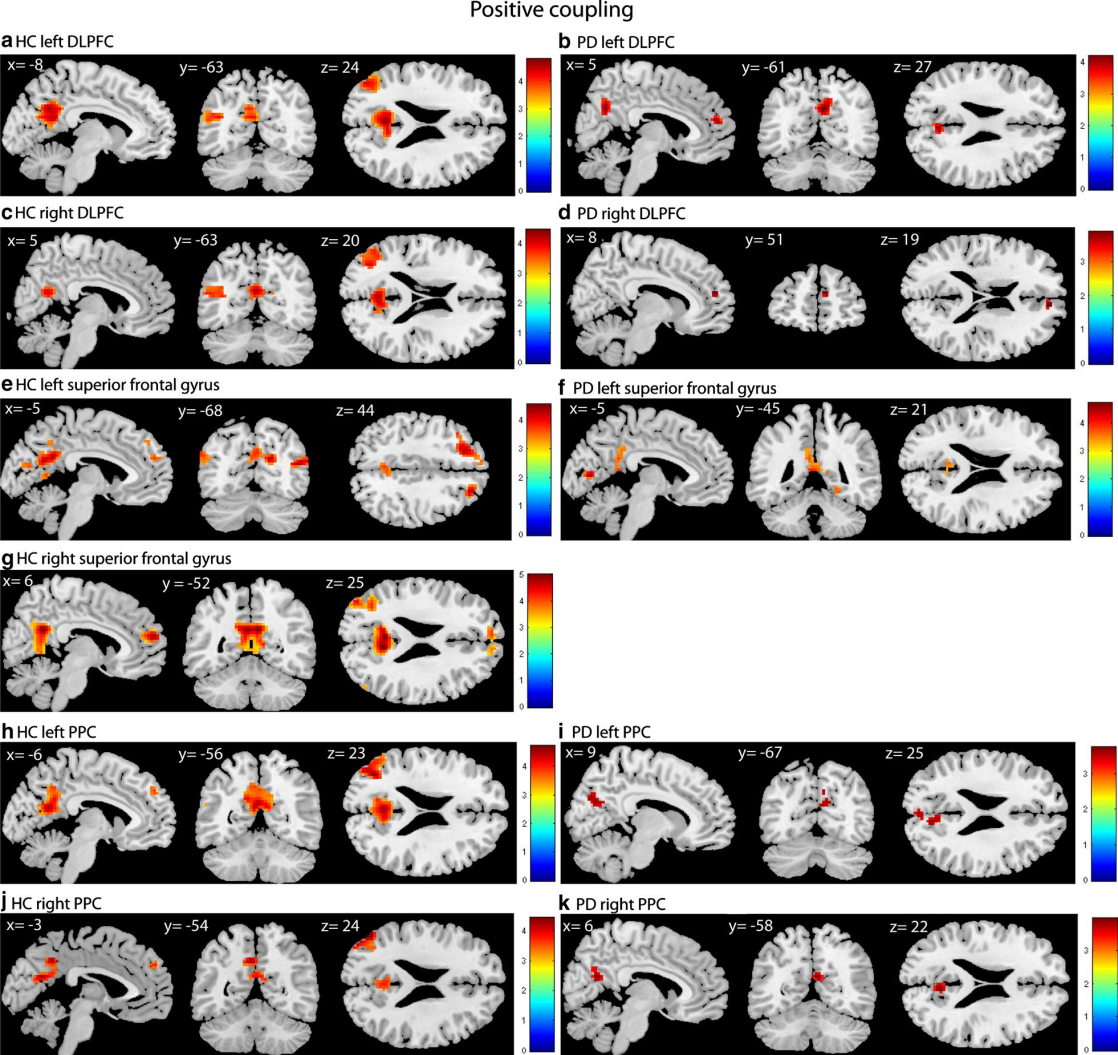
Positive coupling of the DLPFC, superior frontal gyrus and PPC in HC and PD. T-statistic images of positive connectivity in the [successful shift > successful repeat] contrast, corrected for mean RT on shift trials. A voxel-level threshold of p < .001 is used with an extent threshold of 10 voxels. The images are overlaid on ch2better MNI template with MRIcron, coordinates are in MNI space. The *coloured bar* indicates the Z-value

**Fig. 2:**
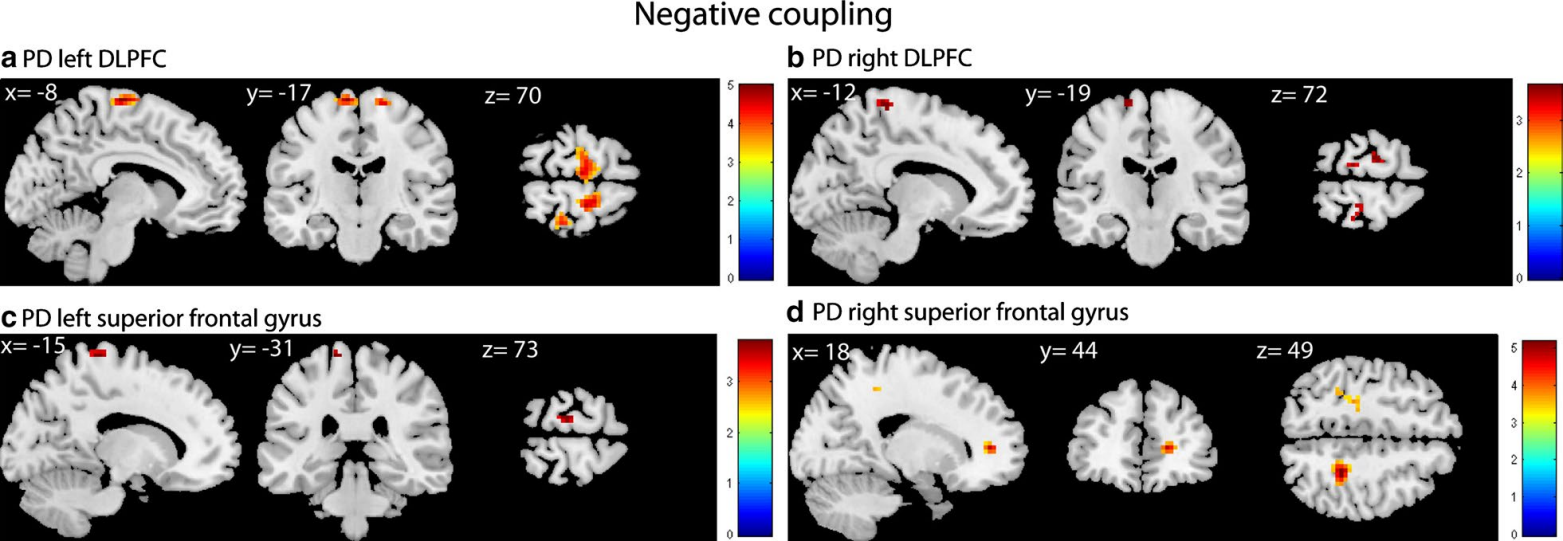
Negative coupling of DLPFC and superior frontal gyrus. T-statistic images of negative connectivity in the [successful shift > successful repeat] contrast, corrected for mean RT on shift trials. A voxel-level threshold of p < .001 is used with an extent threshold of 10 voxels

**Fig. 3:**
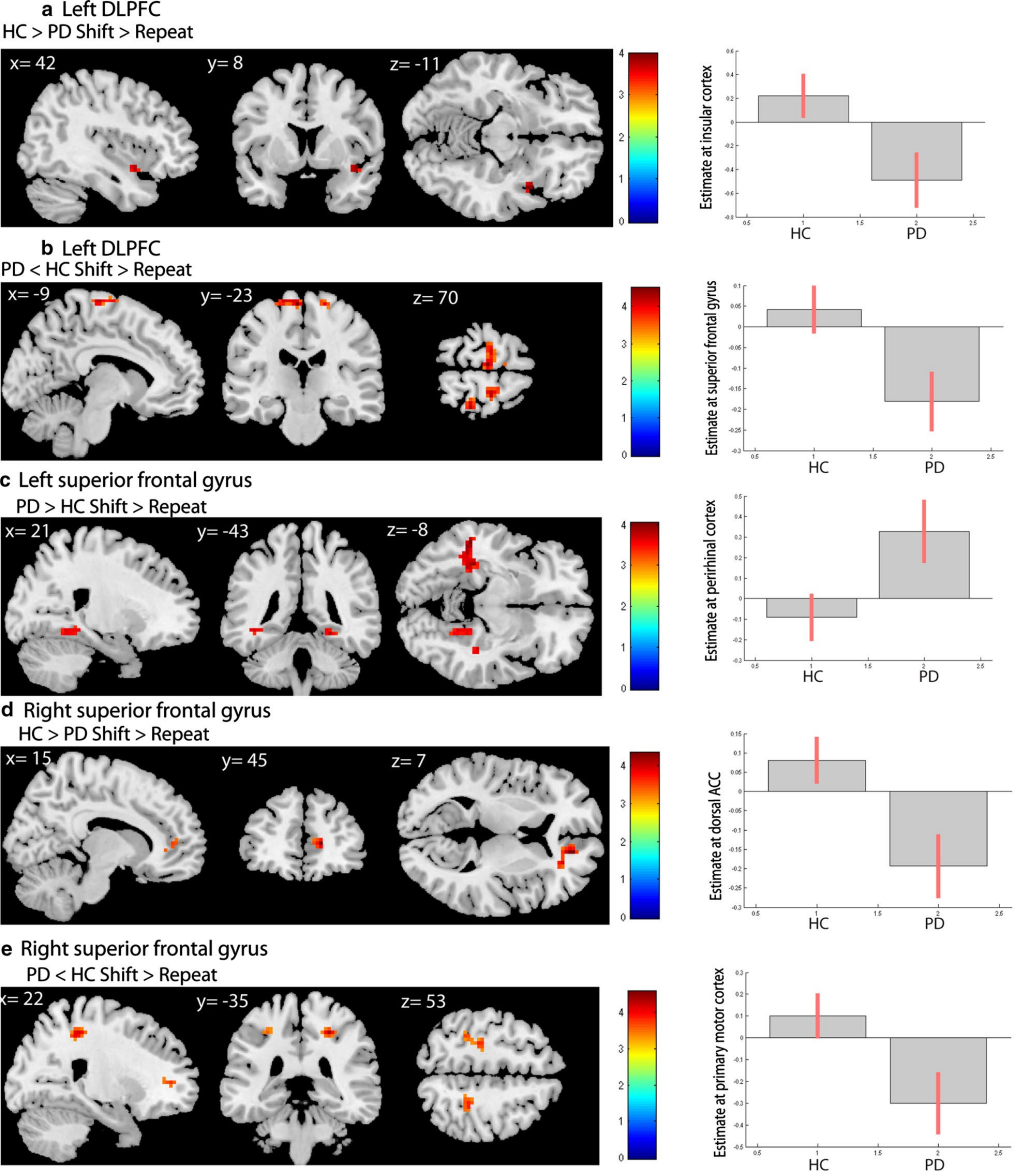
Group interaction results. **a** Left DLPFC HC > PD (masked with main effect HC): increased coupling between the left DLPFC and the right insular cortex in healthy controls compared with PD patients. On the right is depicted the estimate of the right insular cortex peak voxel comparing healthy controls with PD patients. **b** Left DLPFC PD < HC (masked with negative coupling PD): more negative coupling between the left DLPFC and the SFG in PD patients compared with healthy controls. On the right is shown the estimate of the SFG peak voxel comparing healthy controls with PD patients. **c** Left superior frontal gyrus PD > HC (masked with main effect PD): increased coupling between the left SFG and the bilateral perirhinal cortex in PD patients compared to healthy controls. The connectivity difference with the left perirhinal cortex spreads into a larger region encompassing mostly white matter in the left temporal cortex. On the right the estimate of the right perirhinal cortex peak voxel comparing healthy controls with PD patients is shown. **d** Right superior frontal gyrus HC > PD (masked with main effect HC): Greater negative coupling between the right SFG and dorsal ACC in PD patients compared with healthy controls. On the right the estimate of the dorsal ACC peak voxel comparing healthy controls with PD patients is shown. **e** Right superior frontal gyrus PD < HC (masked with negative coupling PD): more negative coupling between the right SFG and the dorsal ACC, primary motor cortex, parietal cortex and precuneus in PD patients compared with healthy controls. On the right the estimate of the primary motor cortex peak voxel comparing healthy controls with PD patients is shown

During set-shifting in the control group, the left DLPFC showed positive coupling with the precuneus, posterior cingulate cortex (PCC) and left angular gyrus (see Fig. 1a). No significant negative coupling was found. In the PD group the left DLPFC showed task-related positive coupling with the precuneus and the right dorsomedial prefrontal cortex (DMPFC) (see Fig. 1b) and negative coupling with the bilateral premotor cortex and pre-supplementary motor area (pre-SMA) (see Fig. 2a). Group comparisons showed that the control group, compared with the PD group, had stronger positive coupling between the left DLPFC and the contra-lateral insular cortex (see Fig. 3a). PD patients, compared with controls, had more negative coupling between the left DLPFC and the SFG and primary motor cortex (see Fig. 3b).

Regarding the right DLPFC, the control group showed positive coupling between this seed region and the left angular gyrus and the PCC (see Fig. 1c). In the PD group the right DLPFC showed positive coupling with the right DMPFC (see Fig. 1d) and negative coupling with the left premotor area (see Fig. 2b). No group differences were found. Table 1 provides an overview of the results.

**Table 1:** Results of the gPPI analyses in the contrast “successful shift > successful repeat”: left DLPFC and right DLPFC

Regions	BA	t-value	Cluster size	Peak voxel coordinates (MNI)		
				X	Y	Z
Left DLPFC						
Positive coupling PD						
Precuneus	7	4.19	66	3	−61	31
Right DMPFC	9	4.17	22	9	50	19
Positive coupling HC						
PCC	23	4.82	361	−3	−55	22
Precuneus	31	4.58		−9	−58	31
PCC	23	4.43		9	−55	19
Left angular gyrus	39	4.36	126	−45	−73	22
Negative coupling PD						
Left SFG	6	4.99	76	−9	−19	73
Right SFG	6	4.58	37	18	−19	70
Right primary somatosensory cortex	2	4.17	14	30	−37	70
Right SFG	6	3.72	26	12	−10	55
Interaction effect positive coupling: HC > PD (masked with main effect of positive coupling HC)						
Right insular cortex	13	3.91	15	42	5	−11
Interaction effect negative coupling: PD < HC (masked with main effect of negative coupling PD)						
Left SFG	6	4.50	57	−6	−22	70
		4.19		−15	−19	73
		3.89		−9	−7	73
Right SFG	6	3.70	13	51	−7	52
Right SFG	6	4.14	24	18	−19	70
Right primary Somatosensory cortex	1	4.14	15	30	−37	70
Right insular cortex	13	3.91	57	42	5	−11
Right DLPFC						
Positive coupling PD						
Right DMPFC	9	3.89	10	9	53	19
Positive coupling HC						
PCC	31	4.47	197	−3	−61	22
	23	4.06		6	−58	19
	31	3.97		−3	−52	28
Left angular gyrus	39	4.01	137	−45	−76	25
		4.00		−36	−70	19
		3.76		−45	−61	16
Negative coupling PD						
Left SFG	6	3.65	11	−18	−22	73

Results of the connectivity analyses: comparison of the PD and HC groups on the successful shift > successful repeat contrast. All areas were significant at an uncorrected threshold of *p* < .001, with an extent threshold of 10 voxels

*HC* healthy controls, *PD* Parkinson’s disease, *BA* Brodmann area

### Functional connectivity SFG

During set-shifting in healthy controls the left SFG showed positive coupling with the precuneus, bilateral angular gyrus, bilateral DMPFC, posterior cingulate cortex and visual cortex (see Fig. 1e). In the PD group the seed region showed positive coupling with the PCC and right perirhinal cortex (see Fig. 1f) and negative coupling with the primary motor cortex (see Fig. 2c). Group comparisons showed greater coupling between the left SFG and the right perirhinal cortex in PD patients compared with controls (see Fig. 3c).

In the control group the right SFG showed positive coupling with the precuneus, bilateral frontal polar cortex, bilateral angular cortex and right lingual gyrus (see Fig. 1g). In the PD group, no positive coupling was found, but the seed region showed negative coupling with the dACC and the primary motor cortex (see Fig. 2d). Group comparisons showed greater positive coupling with the dACC in controls compared with PD patients (see Fig. 3d). PD patients, compared with controls, showed more negative coupling between the seed region and the dACC, primary motor cortex, the precuneus and the PPC (see Fig. 3e). Table 2 displays an overview of the results.

**Table 2:** Results of the gPPI analyses in the contrast “successful shift > successful repeat”: left superior frontal gyrus and right superior frontal gyrus

Regions	BA	t value	Cluster size	Peak voxel coordinates (MNI)		
				X	Y	Z
Left superior frontal gyrus						
Positive coupling PD						
Ventral PCC	23	4.70	107	−9	−76	7
		3.74		6	−76	10
Right perirhinal cortex	36	3.85	20	24	−43	−11
Positive coupling HC						
Precuneus	31	4.55	380	9	−55	25
		4.30		−6	−49	28
		4.22		−6	−61	22
Right angular gyrus	39	4.45	62	51	−73	19
Right frontal cortex	8	4.22	47	24	35	43
Left frontal cortex	8	4.20	111	−21	29	43
		4.08		−36	23	46
		3.38		−9	47	43
Right associative visual cortex	19	4.02	29	30	−88	16
		3.80		24	−94	13
Ventral PCC	23	3.95	29	3	−19	34
Left angular gyrus	39	3.85	59	−45	−79	25
		3.73		−51	−70	31
		3.66		−51	−70	19
Left DMPFC	9	3.78	17	−3	53	25
PCC	30	3.69	16	−9	−58	7
Negative coupling PD						
Left primary motor cortex	4	3.81	11	−15	−31	73
Interaction effect positive coupling PD > HC (masked with main effect of positive coupling PD)						
Right perirhinal cortex	36	3.72	28	24	−43	−11
Right superior frontal gyrus						
Positive coupling HC						
Precuneus	31	5.00	484	6	−52	25
		4.90		−3	−52	25
		4.21		9	−55	13
		3.63	16	3	−34	43
Bilateral anterior prefrontal cortex	10	4.66	194	6	59	19
		4.03		−6	56	28
		3.85		9	47	4
Left angular gyrus	39	4.34	149	−42	−79	28
		4.03		−36	−64	25
		3.96		−54	−61	16
Right lingual gyrus	18	3.78	10	33	−88	1
		3.44		24	−91	4
Right angular gyrus	39	3.58	12	51	−73	19
Negative coupling PD						
Right dACC	32	4.75	22	18	44	7
Left primary motor cortex	4	4.04	28	−30	−25	52
		3.86		−21	−25	52
Interaction effect positive coupling: HC > PD (masked with main effect of positive coupling HC)						
Right dACC	32	4.34	43	15	44	7
Interaction effect negative coupling: PD < HC (masked with main effect of negative coupling PD)						
Right dACC	32	4.41	56	18	44	7
Left primary motor cortex	4	3.78	26	−27	−28	52
Left parietal cortex	40	3.68		−33	−40	52
Right parietal cortex	7	3.66	11	21	−58	46

Results of the connectivity analyses: comparison of the PD and HC groups on the successful shift > successful repeat contrast. All areas were significant at an uncorrected threshold of *p* < .001, with an extent threshold of 10 voxels

*HC* healthy controls, *PD* Parkinson’s disease, *BA* Brodmann area

### Functional connectivity PPC

In both the controls and PD patients (see Fig. 1i), the left PPC showed positive coupling with the precuneus. In the healthy controls the seed region also displayed positive coupling with the left angular gyrus, DLPFC, and DMPFC (see Fig. 1h). No negative coupling was found in either group. In addition, no significant group differences were found.

The right PPC showed positive coupling with the precuneus in both the PD (see Fig. 1k) group and the control group. In the healthy controls the seed region also showed positive coupling with the left angular gyrus and the DMPFC (see Fig. 1j). We found no negative coupling and no group differences for the right PPC seed. Table 3 displays an overview of the results.

**Table 3:** Results of the gPPI analyses in the contrast “successful shift > successful repeat”: left PPC and right PPC

Regions	BA	t-value	Cluster size	Peak voxel coordinates (MNI)		
				X	Y	Z
Left PPC						
Positive coupling PD						
Precuneus	31	3.61	77	3	−61	22
Positive coupling HC						
Left angular gyrus	39	4.73	157	−45	−73	25
		4.56		−39	−67	22
		3.80		−57	−61	16
Precuneus	31	4.62	332	−3	−58	22
		4.04		−12	−55	28
		3.92		−12	−64	22
Left DMPFC	9	3.75	30	−6	50	37
Left DLPFC	9	3.64	157	−15	44	37
Right PPC						
Positive coupling PD						
Precuneus	31	3.96	36	6	−58	22
Positive coupling HC						
Left angular gyrus	39	4.42	79	−51	−70	28
		4.10		−45	−79	25
		3.48		−39	−67	25
Precuneus	31	4.20	138	−3	−64	22
		4.16		−6	−52	40
Left DMPFC	9	3.67	10	−3	53	34

Results of the connectivity analyses: comparison of the PD and HC groups on the successful shift > successful repeat contrast. All areas were significant at an uncorrected threshold of *p* < .001, with an extent threshold of 10 voxels

*HC* healthy controls, *PD* Parkinson’s disease, *BA* Brodmann area

## Discussion

This study investigated differences in task-related functional connectivity between early-stage PD patients and matched healthy controls, using a simple feedback-based set-shifting paradigm. Main effects showed coupling between nearly all seed regions and various key areas involved in cognitive functions, such as the precuneus, the angular gyrus, and the DMPFC in both the PD group and the healthy controls. Overall, PD patients, compared with controls, showed less positive functional connectivity, or more negative functional connectivity, between the seed regions and task-relevant network areas. These findings are in accordance with a recent working memory study in the same study sample [24], and suggest that in early stage PD the normal communication between different task-related brain regions is disrupted during task-performance. We hypothesize that dopamine depletion results in an altered synchronization between task-related brain areas, by either diminishing positive functional connectivity or a maladaptive negative functional connectivity. We propose that the hyper-activation of the individual task-related brain areas that we found in our previous study [15] is a form of compensation for the disrupted functional connectivity of the task-related network.

We found decreased positive functional connectivity between the left DLPFC and the right insular cortex in early stage PD patients when compared with controls. Sridharan et al. [26] argue that the right insular cortex is important for switching between a network that becomes active during rest (i.e. default mode network) and a network that becomes active while performing cognitive tasks (i.e. central executive network). Our data suggest that the left DLPFC in PD patients is less well connected with this important regulatory brain area. In addition, the PD patients showed a decrease in positive coupling of the left SFG with the dACC and an increase in negative coupling of the right SFG with the right dACC. The dACC is connected with the SFG, especially the pre-SMA, and these areas together are important for error detection. In addition, the dACC interacts with the DLPFC and together with the pre-SMA, these three areas are likely involved in cognitive control functions [27].

We found increased negative functional connectivity of the left DLPFC with the SFG in PD patients. This latter area is essential for the planning of movement and cognition, and is normally connected with the DLPFC [28, 29]. Rowe and colleagues found positive coupling between the prefrontal cortex and the pre-SMA in healthy individuals during an attention-to-action task, while this coupling was absent in PD patients [30]. Together, these findings suggest that in PD these important functional connections of the DLPFC with the SFG are disrupted. In addition, in PD compared with controls, the right SFG showed increased negative coupling with left motor cortex. We speculate that the increased negative coupling between the right SFG and the primary motor cortex, might contribute to the increased reaction times during both successful shift and successful repeat trials that we described in our previous article [15].

We found no between-group differences in task-related functional connectivity when using the left and right PPC as seed regions. In our previous study, we found that the parietal cortices displayed hyper-activation, and combined with our present findings, this suggests that the function of the parietal cortex is still relatively preserved in our patient sample, and might be less influenced by the dopaminergic depletion, in contrast to the frontal lobes [2, 3], possibly as a result of the early stage of the disease in our PD patients.

Previous studies have shown that, due to the PD-related pathology, neuronal cell assemblies desynchronize [31], which can be measured as a decrease in positive, or increase in negative, functional connectivity at the level of neuronal oscillations and of brain activation, in rest [18–20, 32], during motor tasks [23, 33], and during cognitive task performance [24]. Our results are line with these previous findings, and suggest that the initial striatal dopaminergic depletion in early stage PD results in a disrupted task-related functional connectivity between neuronal assemblies. We postulate that the disruption in task-related functional connectivity can be compensated for by hyper-activation of the individual brain areas, thereby forestalling cognitive decline. We speculate that when this hyper-activation can no longer compensate for the disrupted connectivity between neuronal assemblies, the hyper-activation will convert into hypo-activation and the set-shifting deficits will become apparent at the behavioural level.

### Strengths and limitations

This study is the first to explore changes in functional connectivity during a set-shifting task in PD patients. The differences in connectivity that we report cannot be attributed to behavioural differences, as we based our contrast on the correctly answered items only. Furthermore, we studied, to our knowledge, the largest group of unmedicated PD patients during a set-shifting task, thereby excluding the potential confounding effect of dopamine replacement therapy, and used a simple feedback-based paradigm to reduce the influence of other cognitive constructs on task performance and neural activation. However, these methodological strengths also make it difficult to compare our results with previous studies. To be maximally sensitive to small, yet meaningful results in this rare population of a cognitively intact group of early stage and medication-free PD patients, we report our results at an uncorrected threshold; to diminish the risk of false positive findings we used a priori defined regions of interest and raised our voxel-level significance threshold from p = .05 to .001 with an extent threshold of 10 voxels. It remains important that our results are replicated before being able to make any definite statements.

## Conclusions

We investigated task-related functional connectivity changes in unmedicated early stage PD patients during a feedback-based set-shifting task. In conclusion, we found altered coupling between seed regions and task-relevant interconnected network areas in PD patients, when compared with controls. These results, together with our previous finding of intact behavioural performance and hyper-activation, support the hypothesis that in PD there is disrupted functional connectivity between task-related brain areas. To further expand our understanding of this process, longitudinal studies should be performed to see how task-related functional connectivity and activity change over time, how they are modified by dopamine replacement therapy, and how these modulations relate to cognitive performance.

## Methods

### Participants

Twenty-two early stage, non-demented PD patients who were not using dopamine replacement therapy and 40 healthy controls participated in this study. Prior to the analyses a number of participants was excluded, due to (1) presence of a comorbid psychiatric disorder (one patient), (2) scanner failure (one patient, one control), more than 3 mm/degrees of movement while performing the task (two controls), (3) extremely low scores on task performance (more than two standard deviations from the median) when compared within the own group (two patients; two controls). This resulted in a total of 53 subjects; 18 PD patients (mean age 59.7 ± 10 years) and 35 healthy controls (mean age 56.7 ± 10 years). All patients were recruited from the movement disorders outpatient clinic of the VU University medical centre (VUmc) in Amsterdam and were diagnosed using the UK Parkinson’s Disease Society Brain Bank criteria for idiopathic Parkinson’s disease [34]. The healthy controls were matched at the group level with the PD patients on age, gender, education and handedness. Education level was measured in 7 levels ranging from 1 (no finished education) to 7 (university training). Exclusion criteria to take part in this study for both groups were current psychiatric or neurological disorders other than PD, a Beck Depression Inventory (BDI) score >15 and a Mini Mental State Examination (MMSE) score <24. Written informed consent was obtained according to the declaration of Helsinki from all participants after reading the protocol, which was reviewed and approved by the medical ethical committee of VUmc (reference number: 2008/145).

### Demographic and clinical characteristics and behavioural performance

As described in our previous study, the groups did not significantly differ in age (*p* = .24), gender (*p* = .78), or handedness (*p* = .56) and there was no difference in MMSE scores (*p* = .23). Patients and controls were similarly highly educated: the median education level for patients was 6 (range 2–7) and for the controls 6 (range 3–7), p = .81. The Beck Anxiety Inventory [BAI, median (range) PD group: 4 (0–16), controls: 0 (0–11)] and the Beck Depression Inventory [BDI median (range) PD group: 4.5 (0–11), controls 0 (0–10)] scores were significantly higher (*p* < .001 and *p* = .01, respectively), but clinically irrelevant, in the PD group compared with the control group. For the PD patients the mean UPDRS was 22 and the median Hoehn and Yahr stage 2. PD patients made more errors during repeat trials (HC 0.72 %; PD 2.2 %, *p* = .004) but not set-shift trials (HC 0.36 %; PD 0.5 %, *p* = .36), and had longer reaction times on both the shift (HC 902 ± 212 ms; PD 1083 ± 336 ms, *p* = .02) and repeat trials (HC 822 ± 200; PD 1019 ± 283, *p* = .01). For further detail see [15].

### Set-shifting task

An arrow was presented on a screen outside the MRI scanner that was visible to the participants via a mirror attached to the head coil. The arrow appeared either on the right or the left side of a fixation cross, and was pointing up or down. Depending on the feature of the stimulus that was relevant at the moment of presentation, participants had to either indicate its location (right or left of the fixation cross) or direction (pointing up or down) using an MRI compatible response box (Cambridge Research Systems Ltd., UK) with four buttons (left, right, top and bottom) which were arranged in a diamond shape. The stimulus was presented for a maximum of 4000 ms, but was terminated upon a button press. When no response was given within this time window, a red screen appeared, indicating a time-out. Each button press was followed by a feedback screen with a fixed duration of 2000 ms, indicating whether the response had been correct (green screen), or incorrect (red screen). Based on the behavioural response made by the participant each trial was classified into one of five categories (see [15]). For this study, we divided the trials into three categories according to the given response, namely (1) “correct repeat” if no set-shift was indicated and the stimulus was correctly categorized according to the current rule, (2) “successful shift” if the preceding feedback signaled a set-shift, and the subsequent stimulus was correctly categorized according to the new rule, and (3) “error trials” were all trials that were not “correct repeats” or “successful shits”. After 4–7 correct repeat trials, a red screen followed a correct response, indicating a set-shift to the other classification rule. The session ended when 20 percent of all trials were correct set-shift trials, and took approximately 20 min to complete.

### MRI data acquisition

Functional MRI data were acquired using a 3.0 T General Electric Signa MR750 MRI scanner at the VUMC in Amsterdam. The scanning included a sagittal three-dimensional T1-weighted scan for anatomical localization (256 × 256 matrix; voxel size = 1 × 0.977 × 0.977 mm; 172 sections). Functional images were obtained using a gradient echo-planar imaging (EPI) sequence (TR = 2100 ms; TE = 30 ms; field of view = 24 cm; 64 × 64 matrix; flip angle = 80°) with 40 ascending slices per volume (3.75 × 3.75 mm in-plane resolution; slice thickness = 2.8 mm; inter-slice gap = 0.2 mm).

### Data analyses

#### Preprocessing and contrasts

As preprocessing, the EPI scans were slice-time corrected, realigned and unwarped, normalized, and smoothed with an 8 mm Gaussian kernel using SPM8 software (http://www.fil.ion.ucl.ac.uk/spm/software/spm8/). We included all trials during the presentation of feedback (with a fixed duration of 2000 ms) in a first level general linear model (GLM) and added the movement parameters as nuisance variables. Our contrast of interest was “successful shift > successful repeat”. We used this contrast to investigate which brain areas became more active when a feedback screen indicated a set-shift instead of a repeat, thereby thus capturing the neural process of the actual set-shift. Because no motor response was required while processing the feedback, this contrast was not contaminated with motor activity.

### PPI analysis

We assessed the task-related functional connectivity of the bilateral DLPFC, bilateral PPC, and bilateral SFG using a generalized form of context-dependent psychophysiological interaction (gPPI) [35] (https://www.nitrc.org/projects/gppi/). A PPI analysis statistically tests in a whole-brain voxel-wise manner whether areas outside the seed region are functionally connected to the seed region during the task [25, 36]. We chose gPPI, instead of the traditional PPI, as it allowed us to model all psychological task conditions into one first-level design, thus improving the model fit [35]. We distinguished positive coupling (i.e. regions in which activity correlated positively with that of the seed region during the task) and negative coupling (i.e. areas in which activity correlated negatively with the seed region during the task). We employed the main effect of positive coupling as an inclusive mask to search for between-group differences in positive coupling, and the main effect of negative coupling to search for between group differences in negative coupling.

**Fig. 4:**
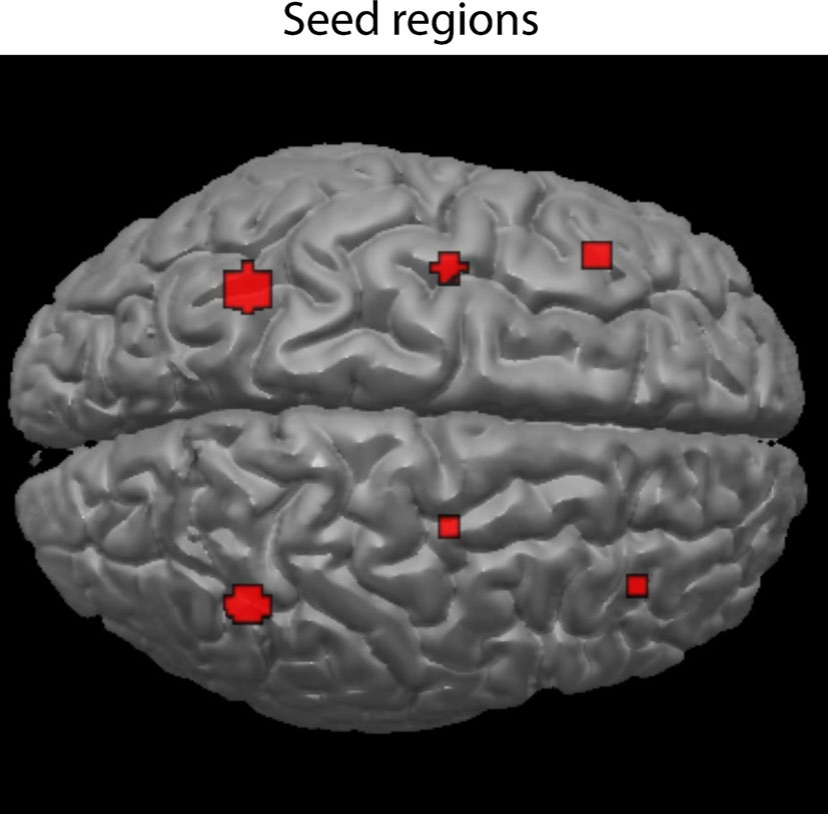
Overview seed regions. Anterior: DLPFC; *right* x = 39, y = 35, z = 31; *left* x = −42, y = 26, z = 31. *Middle* superior frontal gyrus; *right* x = 27, y = −7, z = 58; *left* x = −36, y = −7, z = 64. Posterior: PPC; *right* x = 45, y = −52, z = 49; left x = −33, y = −52, z = 40

The coordinates of the designated seed areas were determined using the peak-voxels of the whole-group activations at second level (DLPFC; right: x = 39, y = 35, z = 31; left: x = −42, y = 26, z = 31; SFG; right: x = 27, y = −7, z = 58; left: x = −36, y = −7, z = 64. PPC; right: x = 45, y = −52, z = 49; left: x = −33, y = −52, z = 40). These coordinates were subsequently used as an initial starting point to find the individual peak-voxel at the first level-contrast “successful shift > successful repeat” within a radius of 5 mm around these previously mentioned coordinates to account for individual variability. The coordinates where manually verified to assure location in the designated area. Next, we constructed six spheres with a 6 (SFG and DLPFC) or 10 mm (PPC) radius around the individually determined peak-voxels, and again used the “successful shift > successful repeat” contrast in the MarsBar toolbox [37] (see Fig. 4).

The first-level models we used thus consisted of the three task conditions (successful shift trials, successful repeat trials, error trials), the time course of the seed of interest, three PPI terms (i.e. the three task conditions convoluted with the time course of the seed region), and six movement parameters. For each seed region, we constructed a separate first level GLM. We only used the PPI terms and our contrast of interest was “successful shift > successful repeat”.

For each of the six seed-regions, a second-level analysis was performed to assess between group differences on the “successful shift > successful repeat” PPI contrast, while employing an independent samples *t* test to compare the controls and PD patients. Because in our previous study [15] the PD patients had an increased RT on the successful shift trials, we included these in the second level analyses as a covariate. Because this is the first study to explore task-related functional connectivity in a group of unmedicated PD patients, we report all results at a voxel-level threshold of *p* = .001, with an extent threshold of 10 voxels.

## Abbreviations

PD: Parkinson’s disease
PPC: posterior parietal cortex
DLPFC: dorso-lateral prefrontal cortex
DMPFC: dorso-medial prefrontal cortex
VLPFC: ventro-lateral prefrontal cortex
SFG: superior frontal gyrus
dACC: dorsal anterior cingulate cortex
pre-SMA: pre-supplementary motor area
WCST: wisconsin card sorting task
fMRI: functional magnetic resonance imaging
MMSE: mini-mental state examination
SCID-I: structured clinical interview for DSM-IV axis-I disorders
BDI: Beck depression inventory
BAI: Beck anxiety inventory
UPDRS: unified Parkinson’s disease rating scale
HC: healthy controls
(g)PPI: (generalized form of context-dependent) psycho-physiological interaction
